# Occult Langerhans Cell Histiocytosis Presenting with Papillary Thyroid Carcinoma, a Thickened Pituitary Stalk and Diabetes Insipidus

**DOI:** 10.1155/2016/5191903

**Published:** 2016-08-30

**Authors:** Michael S. Gordon, Murray B. Gordon

**Affiliations:** ^1^Division of Endocrinology, Diabetes and Hypertension, Brigham and Women's Hospital, Harvard Medical School, Boston, MA, USA; ^2^Division of Endocrinology, Allegheny Neuroendocrinology Center, Allegheny General Hospital, Drexel University College of Medicine, Pittsburgh, PA, USA

## Abstract

Etiologies of a thickened stalk include inflammatory, neoplastic, and idiopathic origins, and the underlying diagnosis may remain occult. We report a patient with a thickened pituitary stalk (TPS) and papillary thyroid carcinoma (PTC) whose diagnosis remained obscure until a skin lesion appeared. The patient presented with PTC, status postthyroidectomy, and I^131^ therapy. PTC molecular testing revealed BRAF mutant (V600E, GTC>GAG). She had a 5-year history of polyuria/polydipsia. Overnight dehydration study confirmed diabetes insipidus (DI). MRI revealed TPS with loss of the posterior pituitary bright spot. Evaluation showed hypogonadotropic hypogonadism and low IGF-1. Chest X-ray and ACE levels were normal. Radiographs to evaluate for extrapituitary sites of Langerhans Cell Histiocytosis (LCH) were unremarkable. Germinoma studies were negative: normal serum and CSF beta-hCG, alpha-fetoprotein, and CEA. Three years later, the patient developed vulvar labial lesions followed by inguinal region skin lesions, biopsy of which revealed LCH. Reanalysis of thyroid pathology was consistent with concurrent LCH, PTC, and Hashimoto's thyroiditis within the thyroid. This case illustrates that one must be vigilant for extrapituitary manifestations of systemic diseases to diagnose the etiology of TPS. An activating mutation of the protooncogene BRAF is a potential unifying etiology of both PTC and LCH.

## 1. Introduction

Etiologies of a thickened pituitary stalk (TPS) include inflammatory, neoplastic, and idiopathic origins, and the underlying diagnosis may remain occult or be a diagnosis of exclusion [1]. We report a case of a patient with TPS and papillary thyroid carcinoma (PTC) whose diagnosis remained obscure until a skin lesion appeared.

## 2. Case Report

**Figure 1:**
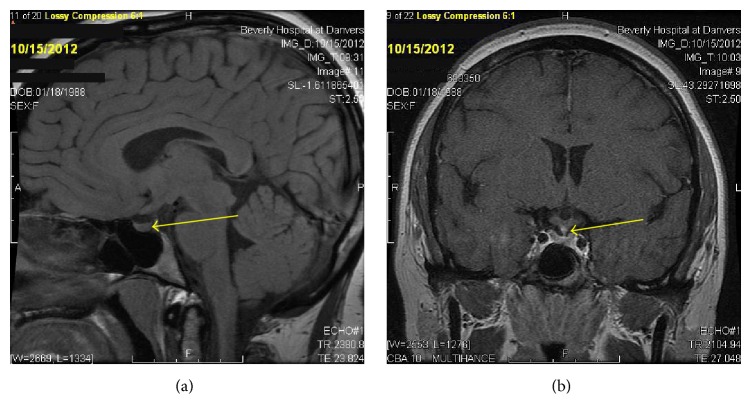
MRI shows absence of posterior pituitary bright spot (a) and thickened pituitary stalk (b).

**Figure 2:**
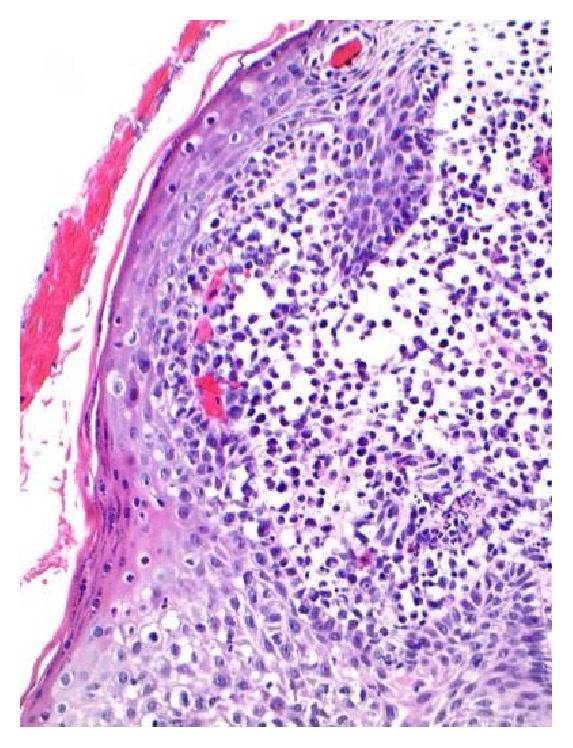
Vulvar biopsy showing Langerhans cell proliferation extending to the tissue edges. Heterogeneous collections of Langerhans cells with eosinophils, neutrophils, small lymphocytes, and histiocytes are demonstrated.

**Figure 3:**
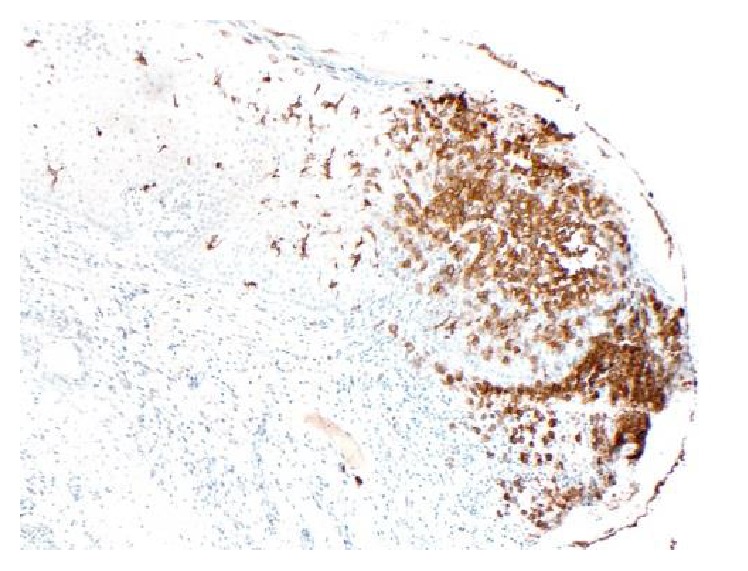
Immunoperoxidase stain of vulvar biopsy showing expression of CD1 consistent with LCH.

The patient presented with PTC at age 22, status postthyroidectomy, and I^131^ therapy. She had a 5-year history of polyuria/polydipsia. Overnight dehydration study confirmed diabetes insipidus; 24 hr urine volume was 12.1 liters. Polydipsia and polyuria responded to desmopressin nasal spray. MRI revealed TPS with loss of the posterior pituitary bright spot (Figures 1(a) and 1(b)). Evaluation showed hypogonadotropic hypogonadism (E2 17 pg/mL, LH 0.7, and FSH 1.2 mIU/mL) and IGF-1 34 ng/dL (SDS −3.2) consistent with growth hormone deficiency. Chest X-ray and ACE levels were normal. A skeletal survey was unremarkable. The survey included x-rays of long bones, pelvis, and skull which are the most frequent sites of bony involvement in Langerhans Cell Histiocytosis (LCH) [2]. Evaluation for a germinoma was negative: normal serum and CSF beta-hCG, alpha-fetoprotein, and CEA. Three years later, the patient developed vulvar labial lesions that responded to courses of oral prednisone. Initial biopsy showed acute and chronic inflammation and repeat biopsy after spread to the inguinal region was consistent with LCH (Figures 2 and 3). A PET CT scan did not show any other sites of involvement with LCH. The thyroid pathology was sent for reanalysis and showed 2 foci of follicular variant of PTC in a background of chronic thyroiditis as well as Langerhans cells (positive staining for S-100, CD1a, langerin, and CD68), consistent with concurrent LCH, PTC, and Hashimoto's thyroiditis within the thyroid. PTC molecular testing revealed BRAF mutant (V600E, GTC>GAG). She was treated with high dose prednisone with transient improvement in TPS and skin, with no change in pituitary function.

## 3. Discussion

Primary etiologies of TPS include inflammatory (lymphocytic hypophysitis/lymphocytic infundibuloneurohypophysitis, sarcoidosis, Wegener's granulomatosis, TB, and Whipple's disease), neoplastic (craniopharyngioma, germinomas, metastases including lymphoma, pituitary adenomas, other primary CNS tumors including glioma, and pituicytoma), Langerhans Cell Histiocytosis (mixed inflammatory/neoplastic classification), and congenital anomalies (pituitary hypoplasia, Rathke's cleft cyst) [2].

Endocrine manifestations of multisystem LCH in adults include diabetes insipidus (DI) with a prevalence of 40–94%, making it the most common disease-related permanent consequence [3, 4]. Fifty-one percent of patients presenting with DI will develop other LCH manifestations within one year [5]. Almost all have loss of the posterior pituitary bright spot on MRI. TPS is observed in 71% at time of diagnosis of DI. Anterior pituitary hormone deficiencies are observed in up to 20% of LCH patients and are almost always associated with DI. If DI led to the evaluation of the anterior pituitary, anterior pituitary abnormalities are seen in 67%. The deficiencies are generally permanent and not affected by LCH directed therapy [6], as in our case. Growth hormone deficiency is most frequently seen with a prevalence of 60% in patients with LCH and DI [7]. Gonadotropin deficiency is the second most common with a prevalence of 55% in patients with LCH and DI [7]. TSH deficiency is seen in 13% of LCH with DI patients and ACTH deficiency in 8% [7]. Direct thyroid involvement with LCH is very rare and can present as localized LCH lesions or part of multisystem disease [8].

Several cases of the coexistence of LCH and PTC have been described as in our patient [9, 10]. About half of both LCH and PTC patients are found to have somatic activating mutations of the protooncogene BRAF, suggesting an etiologic link between these two disorders [9, 11] although the role of the BRAF mutation in the apparent association between LCH and PTC is unknown [12]. One possible mechanism of two identical somatic mutations arising in two different tumors is that BRAF mutations can be found in tumor-surrounding nontumoral tissues. This has specifically been described in PTC [13] as well as melanomas where BRAF mutations were present in normal skin distant from nevi [14]. It should be noted that both LCH and PTC were present within the thyroid in our case. Patients with LCH and BRAF mutations should be monitored for PTC.

## 4. Conclusion

A TPS may be a manifestation of a systemic disease that may not appear for months to years after the initial presentation. An activating mutation of BRAF may explain the possible association of PTC and LCH.
